# Low Formaldehyde Binders for Mineral Wool Insulation: A Review

**DOI:** 10.1002/gch2.202100110

**Published:** 2022-01-12

**Authors:** Thomas M. Bennett, John F. Allan, Jennifer A. Garden, Michael P. Shaver

**Affiliations:** ^1^ School of Natural Sciences Department of Materials The University of Manchester Manchester M13 9PL UK; ^2^ Superglass Insulation Ltd. Thistle Industrial Estate, Kerse Rd Stirling FK7 7QQ UK; ^3^ Sustainable Materials Innovation Hub Henry Royce Institute The University of Manchester Oxford Road Manchester M13 9PL UK; ^4^ School of Chemistry University of Edinburgh Edinburgh EH9 3FJ UK

**Keywords:** formaldehyde scavengers, insulation, mineral wool, phenolic resins, thermoset

## Abstract

Insulating materials are ubiquitous in a built environment and play a critical role in reducing the energy consumed to maintain habitable indoor environments. Mineral wool insulation (MWI) products, including glass, stone, and slag variants, are the most widely used class of insulating materials in Europe and account for more than 50% of the total market by volume. MWI typically consists of two key components: a mesh of inorganic fibers that are several micrometers in diameter, and an organic thermosetting adhesive commonly referred to as the “binder.” Traditional phenol‐formaldehyde‐urea (PFU) binders used in the manufacture of MWI are increasingly being scrutinized for the formaldehyde released during their manufacture and service lifetime. The recent classification of formaldehyde as a carcinogen by various safety organizations has accelerated a paradigm shift within the industry toward alternative binder technologies that minimize or indeed eliminate formaldehyde emissions. This review examines more recent strategies for achieving low‐ or zero‐added formaldehyde binders for MWI, with a particular focus on the patent literature. The chemistry underpinning traditional PFU binders is presented and compared to new strategies involving scavenging molecules that decrease formaldehyde emissions, as well as zero‐added formaldehyde binder technologies such as polyester, Maillard, and epoxide thermosets.

## Graphical abstract

Traditional phenol‐formaldehyde‐urea binders used in mineral wool insulation products release formaldehyde during their manufacture and service lifetime. This review examines recent strategies for achieving low‐ or zero‐formaldehyde binders, such as through the use of scavenging molecules that decrease formaldehyde emissions, as well as formaldehyde free binders including polyester, Maillard, and epoxide thermosets.

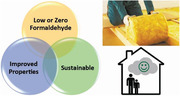

## 1 Introduction

**Figure 1:**
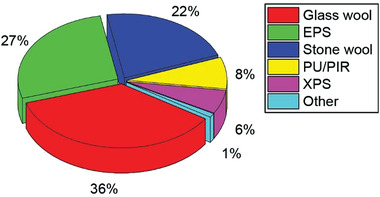
Europe thermal insulation market by volume in 2014. Expanded polystyrene foam is abbreviated as EPS. Polyurethane/polyisocyanurate is abbreviated as PU/PIR. Extruded polystyrene is abbreviated as XPS. Data sourced from ref. ^[^ ^3^ ^]^.

The primary role of insulating materials in society is to passively reduce unwanted energy emissions. This is predominantly thermal energy, yet their use extends to other associated areas including electrical, acoustic, fire resistance, radiation, and moisture protection. Insulation products therefore provide a vital role in minimizing global energy consumption and reducing carbon emissions.^[^ ^1^ ^]^ In 2019, the total European market for thermal insulation products was estimated to be 269 million m^3^ (8.5 million metric tons).^[^ ^2^ ^]^ Fibrous inorganic materials—namely, mineral wools comprised of glass or stone—are one of the most effective and well‐established classes of insulation materials globally and represent an ≈58% market share by volume in Europe (**Figure** **1**).^[^ ^3^, ^4^ ^]^ Mineral wool insulation (MWI) products have seen widespread industrial use due to their high thermal and acoustic insulation properties, non‐flammability, low cost, and ease of installation.

MWI products are predominantly installed in the external walls, floors, and roofs of residential and commercial buildings, with building and construction sectors accounting for an estimated 87% of the total market demand.^[^ ^3^ ^]^ The purpose of this review is to provide a detailed summary of recent developments in technologies underlying the production of MWI products, focusing in particular on strategies to reduce, or eliminate, the quantity of formaldehyde released by the binder before and during their service lifetimes. The reader is directed to several review articles that provide comprehensive overviews of the manufacture, use, and properties of MWI and other types of insulation materials not considered here.^[^ ^5^, ^6^, ^7^, ^8^ ^]^

## 2 Mineral Wool Insulation: Challenges

**Figure 2:**
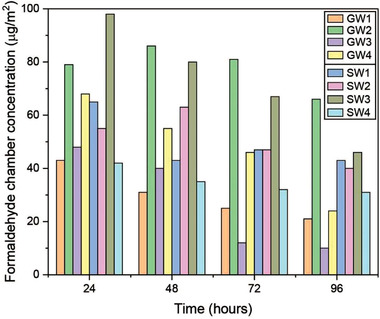
Formaldehyde concentrations in a 1 m^3^ test chamber after test times of 24–96 h. Samples were obtained from four different glass wool (GW) and stone wool (SW) insulation products. The chamber conditions were temperature = 23 °C, relative humidity = 50%, air exchange rate = 1 h^−1^ and loading factor = 1 m^2^ m^−3^. Adapted with permission.^[^ ^10^ ^]^ Copyright 2018, The Authors, published by Elsevier.

One of the biggest technical challenges faced by the MWI industry over the past half century has been the need to reduce the release of various hazardous volatile organic compounds (VOCs) from its products. Release can occur during both the manufacturing process and throughout the service lifetime, contaminating the atmosphere breathed by plant workers, building inhabitants, and users. These VOCs predominantly originate from the resins used as binders, traditionally composed of phenol formaldehyde (PF), and urea. Examples of VOCs include ammonia and phenol, yet formaldehyde has received the most interest due to its designation as carcinogenic to humans by the International Agency for Research on Cancer (IARC) and the World Health Organization (WHO).^[^ ^9^ ^]^ The concentration of formaldehyde emitted from MWI products has been estimated using test chambers; much of this literature has been recently summarized by Salthammer and co‐workers^[^ ^10^, ^11^ ^]^ (**Figure** **2**). Nevertheless, it is important to note that formaldehyde emissions cannot be completely avoided in new home constructions as many other common building products (e.g., woods, paints, wallpaper, etc.) also release low amounts of formaldehyde.^[^ ^12^ ^]^

In response to these increasingly stringent regulations there has been a concerted effort by mineral wool manufacturers over the past few decades to reduce or eliminate formaldehyde emissions from their products. Customer perception and marketability has also become a strong motivator. This effort has resulted in a flurry of patent filings, concerning both methods of scavenging formaldehyde molecules that are released during and after the curing of PF resin binders, and the development of novel binders that are not prepared from formaldehyde. In the following stages of this article, the manufacturing process of MWI products is first described, along with a detailed description of the chemical reactions underlying traditional PF resin binders. A thorough summary of the academic and patent literature in the areas of both formaldehyde scavengers in PF resin binders and formaldehyde free binders is then presented, and their modes of action are discussed. We note here that caution must be taken when interpreting information contained in patents, which often seek to disguise the true core of the invention from competitors. In each case we have paid particular attention to the experimental examples and list of novel claims contained within the patents discussed herein.

## 3 Manufacture of Mineral Wool Insulation Using Phenol‐Formaldehyde Resin Binders

**Figure 3:**
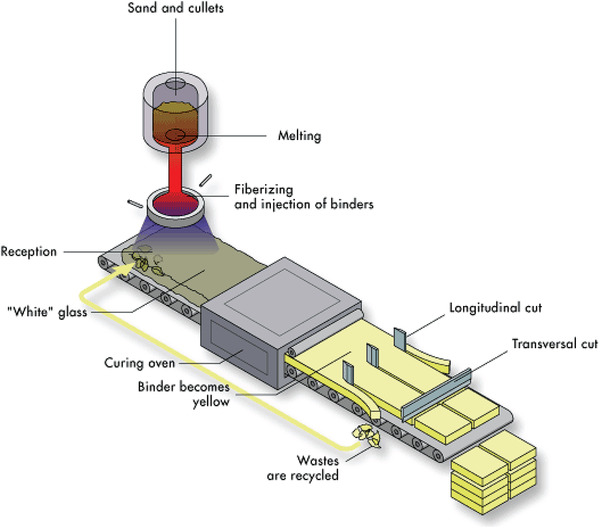
Production process for glass MWI. Reproduced with permission.^[^ ^13^ ^]^ Copyright EURIMA.

Mineral wool manufacture consists of the following stages: raw material preparation, melting, fiberization of the melt, binder application, product mat formation, curing, cooling, and product finishing. Taking glass wool as an example, the manufacturing process begins with the weighing and mixing of the raw materials, followed by transportation to a furnace, and smelting at temperatures >1000 °C (**Figure** **3**). Production typically requires raw materials comprising up to 85% recycled glass (referred to as “cullet”) with the remainder made up by sand, limestone, soda, borax, and/or dolomite. The molten composition is then transported to a rotating disk or “spinner” which consists of a flywheel containing thousands of micrometre sized holes. This spinner is composed of high grade metal alloys and typically rotates at a frequency of several thousand revolutions per minute (rpm). As the molten minerals move into the spinner they are drawn through these holes and fiberizing takes place. Upon exiting the flywheel, with the assistance of a downwards airflow, the minerals cool and solidify, forming long, fine fibers which combine to produce a “wool”. It is at this point in the process that the binder mixture is applied via spraying.

**Figure 4:**
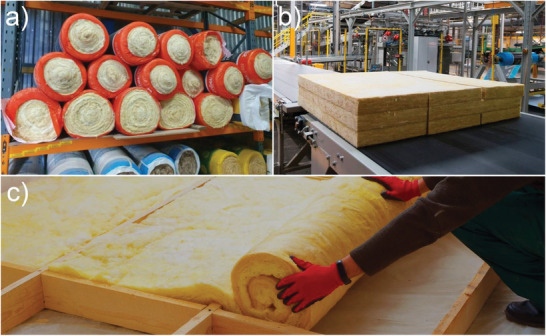
a) Packaged rolls of glass MWI. b) Cut slabs of glass MWI prior to packaging. c) An installer unrolling a MWI product at a construction site.

The binder mixture, containing a thermosetting adhesive, settles at the junction points of the mineral fibers due to physical forces such as surface tension and gravity. The sprayed mineral wool is then transported into a collecting chamber where water evaporation is accelerated at ≈80 °C before it is moved into curing ovens, allowing the binder to cure at temperatures of 150–250 °C. The cured binder adheres the mineral fibers together, thereby providing the mineral wool with its characteristic mechanical properties such as compressive, tensile and bending strengths. The material is then removed from the ovens and subsequently cut into the desired shape.^[^ ^14^ ^]^ The final step in the production process involves packing the material into either rolls, mats or batts in preparation for transportation (**Figure** **4**).

**Scheme 1:**
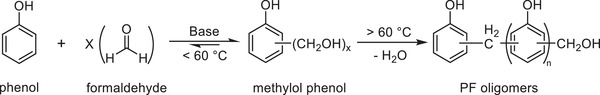
Methylolation reaction of phenol and formaldehyde followed by PF oligomer formation.

The aqueous binder composition is traditionally comprised of the following components: a PF resole, urea, organosilanes, silicones, ammonium sulfate, ammonia, emulsifiers, and water. PF resins are formed through condensation reactions between phenol and formaldehyde. Prior to curing, a resole is typically a mixture of methylol phenols, various oligomers, and residual free phenol and formaldehyde. Depending on the formaldehyde:phenol feed ratio, the resole mixture may contain anywhere between 5 and 15 wt% of residual free formaldehyde.^[^ ^15^ ^]^ On exposure to higher temperatures (e.g., >60 °C), the resole can be cured through further condensation reactions, ultimately leading to the formation of a highly crosslinked, methylene bridged polymeric network (**Scheme** **1**). Most of the unreacted formaldehyde is released during this stage as a VOC. The reader is directed to several book chapters on PF resins for more detailed mechanistic summaries of these chemical reactions.^[^ ^16^, ^17^, ^18^, ^19^ ^]^

The resulting materials possess a range of desirable properties as adhesives, including a high‐modulus, high crosslink density, moderately high glass transition temperature (median value ≈150 °C), along with excellent moisture and heat resistance (**Table** **1**).^[^ ^15^, ^16^, ^18^, ^19^, ^20^ ^]^ Such properties, coupled with their low‐cost, have historically made PF resins ideal for use in the production of MWI in order to achieve a strongly bound product fit for purpose in a range of environments.

**Table 1:** Typical properties of phenolic resins^[^ ^18^, ^19^, ^20^, ^21^ ^]^

Property [units]	Typical values
Cured density [g cm^−3^]	1.24–1.32
Tensile strength [MPa]	24–45
Tensile modulus [GPa]	3–5
Elongation at break [%]	0.3–2.0
Dielectric constant (*D*_k_) (1 Hz)	4–10
Cure shrinkage [%]	0.1
Onset of thermal degradation (*T*_5%_) [°C]	300–360
Glass transition temperature (*T*_g_) [°C]	90–290
Coefficient of thermal expansion [ppm °C^−1^]	16.2–24.7
Water absorption (24 h) [%]	0.2–0.4
Cost [€ kg^−1^] (in 2013)	1.24–1.41

**Scheme 2:**
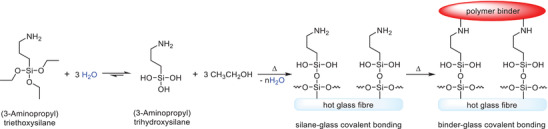
Coupling pathway between mineral fibers and binder via (3‐aminopropyl)triethoxysilane.

Small additions of organosilanes can significantly increase the mechanical strength of mineral wool. This effect is generally more pronounced in binders that are wet and/or that have been aged for prolonged periods (e.g., months). Inexpensive (3‐aminopropyl)triethoxysilane is the most commonly used, although many others have also been reported.^[^ ^22^ ^]^ The organosilane acts as a coupling agent between the mineral fibers and binder, undergoing a series of reactions ultimately resulting in covalent bond formation between glass fiber surfaces (**Scheme** **2**).^[^ ^15^, ^23^, ^24^, ^25^ ^]^

Silicones act as a hydrophobic barrier that enhances the water‐resistant properties of the mineral wool.^[^ ^26^, ^27^ ^]^ Ammonia ensures that the binder has a basic pH, usually between 8 and 10. This pH is necessary to prevent the various oligomers that comprise the binder from precipitating. An oil‐based emulsifier is added to the binder mixture mainly to reduce the dust produced during manufacture, as well as improving the hydrophobicity of the mineral wool. Water is added as a diluent to reduce the viscosity of the binder, improving its flow properties and processability. Ammonium sulfate acts as a latent hardening agent by causing the binder to gradually become acidic throughout the curing process. Acidic conditions improve the polymerization reaction and enable a stronger resin to be obtained.^[^ ^15^ ^]^ Addition of ammonium sulfate is also a means of regulating the B‐stage cure time. The curing process of a PF resin can be separated into three main phases: liquid resole (A‐stage), gelled resin (B‐stage), and fully crosslinked resin (C‐stage).^[^ ^28^ ^]^ The B‐stage must be of a sufficient time so as to allow the binder to settle at junction points between mineral fibers before hardening, and typically takes longer at neutral pH conditions.^[^ ^19^, ^29^ ^]^ If the B‐stage is too short, it will lead to pre‐curing of the binder before it reaches the junction points. If the B‐stage is too long, it could result in an incomplete cure reaction. Both cases have a detrimental effect on the mechanical properties of the complete product.

**Scheme 3:**
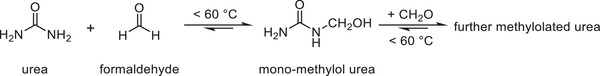
Methylolation reaction between urea and free formaldehyde.

The initial approach to reducing formaldehyde emissions is through the addition of urea, which reacts with formaldehyde in a similar manner to phenol, forming methylol ureas (**Scheme** **3**). These newly formed methylol ureas can participate in the curing reaction, thereby chemically trapping residual free formaldehyde within the polymeric network. Incorporation of urea within the mineral wool binder imparts numerous other advantages such as low cost, improved fire resistance, improved binding strength,^[^ ^30^ ^]^ viscosity reduction and improved non‐punking properties.^[^ ^31^ ^]^ Punking is the phenomenon of slow, flameless burning that can cause decomposition of the cured binder and poses a fire hazard to combustible materials in the vicinity.^[^ ^32^ ^]^ Urea also acts as an extender that serves to lower the percentage of PF resole present in the binder mixture, further reducing formaldehyde emissions via a dilution effect.

**Scheme 4:**
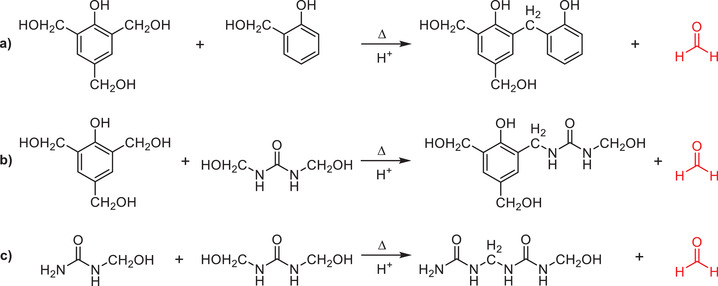
Example pathways for the release of formaldehyde (red) when curing phenol‐formaldehyde‐urea (PFU) resins.

Although the addition of urea significantly reduces the amount of residual free formaldehyde within the binder, it fails to completely eliminate the issue of formaldehyde emissions.^[^ ^33^ ^]^ Furthermore, formaldehyde is produced as a result of cure reactions taking place between various methylolated species (**Scheme** **4**). It has also been observed that methylol ureas are unstable at the temperatures used during the curing reaction, causing a small portion to revert into urea and formaldehyde.^[^ ^34^, ^35^ ^]^ The tendency of methylol ureas to degrade via hydrolysis is also a route by which free formaldehyde can be released from MWI products as a VOC during their service lifetime.^[^ ^36^ ^]^

**Figure 5:**
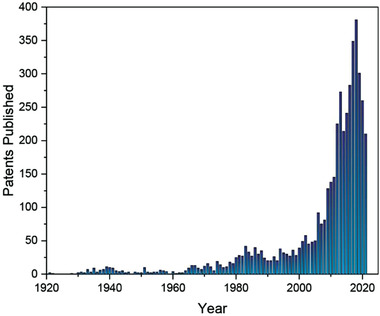
SciFinder search data histogram for number of patents published each year between 1921 and 2021 that contain at least one of the terms “mineral wool insulation”, “glass wool insulation”, or “stone wool insulation”.

These various issues have driven mineral wool manufacturers to develop new methods aimed at further reducing formaldehyde emissions in recent years.^[^ ^37^, ^38^ ^]^ More effective formaldehyde scavenging methods have been developed, often for use in combination with urea, to give PF resin binders with very low formaldehyde emissions. Meanwhile, alternative thermosetting adhesive binder systems have been developed which do not use formaldehyde as a raw material and can therefore be considered “formaldehyde free”, as discussed further below. New binder systems must have a sufficient viscosity to allow ease of processing whilst also curing to provide a solid resin with required mechanical properties. Achieving binders with these characteristics that are also cost effective is an ongoing challenge in the industry. A SciFinder search for the number of patents published each year between 1921 and 2021 that contain at least one of the terms “mineral wool insulation”, “glass wool insulation”, or “stone wool insulation”, serves to highlight the prolific patenting activity that has taken place in this area over the past two decades in particular, many of which are related to novel binder development (**Figure** **5**).

## 4 Methods of Formaldehyde Reduction in PF Resole Binders for Mineral Wool Insulation

### 4.1 Carbamides, Carbonyls, and Imines

**Figure 6:**
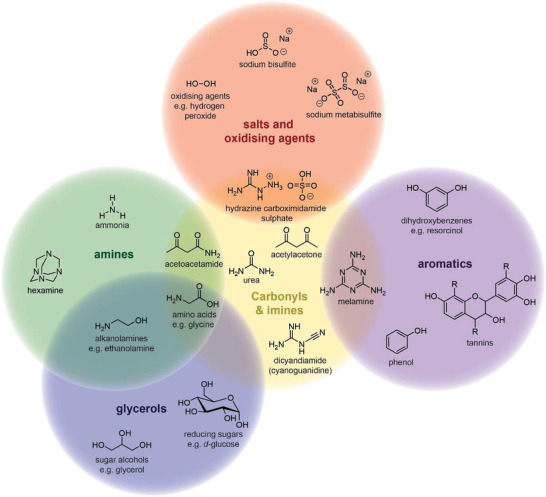
Chemical structures of common formaldehyde scavengers used in PFU binder compositions for MWI.

Industry practice involves the addition of formaldehyde “scavengers” to the PF resole in order to reduce formaldehyde emissions (**Figure** **6**). Some of the most prevalent formaldehyde scavengers are nitrogen‐containing organic compounds soluble within the resole mixture that possess functional groups such as carbamides and/or various other related moieties. Urea is by far the most common formaldehyde scavenger owing to its low‐cost and numerous advantages outlined earlier such as increased binder strength. A limit to the amount of urea added to the resole is observed where further additions lead to a reduction in the binder strength.^[^ ^15^, ^24^ ^]^ Urea is also thought to contribute towards the production of trimethylamine (TMA), which thermally degrades to produce an undesirable “fishy” odor.^[^ ^39^ ^]^ Urea produced from this thermal degradation, along with any residual free urea present in the binder, contributes towards the formation of ammonia which is subsequently released as a VOC.^[^ ^40^ ^]^ Ammonia emissions pose a risk to the environment and may cause eye, nose, and throat irritation in plant workers. Ideally, ammonia emissions are kept as low as possible during curing and must be kept in balance with the release of other VOCs through careful control of the binder formulation.

It has been demonstrated that formaldehyde emissions can be further reduced if the addition of urea takes place in two stages rather than one for the same total quantity of urea.^[^ ^41^, ^42^, ^43^ ^]^ Arenas et al.^[^ ^41^, ^42^ ^]^ speculates that this approach alters the thermodynamics of the cure reaction in a way that incorporates more formaldehyde into the polymer network. Melamine, dicyandiamide, acetoacetamide, and acetylacetone have also all proven effective as formaldehyde scavengers used as alternatives to, or in combination with, urea (Figure 6, yellow circle).^[^ ^44^, ^45^, ^46^, ^47^, ^48^, ^49^ ^]^ These compounds can reduce formaldehyde emissions while also producing stable condensates that are less likely to thermally degrade at cure temperature conditions. Furthermore, melamine has been shown to reduce the formation of TMA. It has also been documented that the addition of a pre‐reacted melamine resin to a phenol‐formaldehyde‐urea (PFU) resin induces a similar effect.^[^ ^50^ ^]^

### 4.2 Hydroxybenzenes

Aromatic hydroxyl compounds such as dihydroxybenzenes (e.g., resorcinol) or tannins can also scavenge formaldehyde (Figure 6, purple circle).^[^ ^51^, ^52^, ^53^, ^54^, ^55^ ^]^ Whilst not possessing any amine or amide groups, the aromatic hydroxyls are able to undergo a methylolation reaction akin to the methylolation of phenol, but are far more reactive.^[^ ^56^ ^]^ Through this process, stable products are formed which can participate in curing reactions, thereby chemically trapping formaldehyde within the polymeric network. It has since shown that binder precursors can also be created by reacting resorcinol with aldehydes other than formaldehyde, namely cyclohexane dicarboxaldehyde and glutaraldehyde (and mixtures thereof), in the presence of a strong base catalyst (e.g., sodium hydroxide).^[^ ^55^ ^]^ These formaldehyde free resins can then be used to substitute a large portion of the existing PF binder, thus acting to reduce formaldehyde by a combination of both scavenging and substitution. Such formulations are reported as being competitive with PFU resins on a cost per performance basis because they involve similar precursors and are also compatible with the urea extension process.

### 4.3 Bisulfites

Sodium/ammonium (meta)bisulfites are also utilized as formaldehyde scavengers in several patents concerning PFU binders for MWI, forming addition products with formaldehyde (Figure 6, red circle).^[^ ^57^, ^58^, ^59^ ^]^ Taylor and Shannon^[^ ^57^ ^]^ claim that a 40–60% reduction in free formaldehyde is observed across a range of binder examples when they contain as little as 5 wt% of sodium or ammonium bisulfite (relative to binder solids). Oven ammonia emissions were also noted as decreasing by 14% in some cases. Furthermore, the addition products did not appear to break down at elevated temperatures (e.g., in the curing oven), thereby indicating that the bisulfite becomes incorporated into the binder during cure.

### 4.4 Alkanolamines

**Scheme 5:**
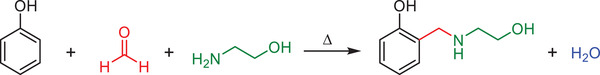
Mannich‐type reaction between phenol, formaldehyde and ethanolamine.

Alkanolamines such as mono‐, di‐, and triethanolamine are examples of formaldehyde scavengers capable of forming condensates with both phenol and formaldehyde (Figure 6, green and blue circles).^[^ ^52^, ^60^, ^61^, ^62^ ^]^ Such alkanolamines are capable of undergoing Mannich‐type reactions with free formaldehyde and free phenol, forming Mannich bases that are thermally stable at cure temperature conditions (**Scheme** **5**). By extension, glycine is used as a formaldehyde scavenger as it also possesses the ability to undergo Mannich‐type reactions.^[^ ^63^ ^]^ Therefore, alkanolamines and glycine possess the advantage of being able to simultaneously reduce ammonia, phenol, and formaldehyde emissions. More recently, Hünig^[^ ^64^ ^]^ reported phenolic resin compositions containing an ethanolamine‐neutralized polyacrylic acid (PAA), which presumably acts as a multisite reactive species that increases crosslink density.

### 4.5 Ammonia

Gabrielson et al.^[^ ^65^ ^]^ proposes the use of ammonia or ammonium hydroxide in order to scavenge free formaldehyde, forming amine compounds such as hexamethylenetetramine, otherwise known as hexamine (Figure 6, green circle). However, a binder system using ammonia or ammonium hydroxide as a formaldehyde scavenger will likely trade a reduction in formaldehyde emissions for an increase in ammonia emissions. One proposed solution to the increased ammonia emissions is through incorporation of a reducing sugar compound, preferably glucose, in conjunction with the ammonia or ammonium hydroxide.^[^ ^66^ ^]^ The aldehyde or ketone present on the reducing form of these sugars reacts with ammonia and thereby decreases ammonia emissions.

### 4.6 Sugars and Carbohydrates

The use of sugars and other similar carbohydrates within binder compositions is not purely limited to the reduction of ammonia emissions (Figure 6, blue circle). Binder compositions also benefit in terms of cost and sustainability from the extension effect provided by various carbohydrates. It is understood that most carbohydrates do not take part in binder reactions until the curing stage, unless allowed to react for prolonged periods of time (e.g., days).^[^ ^67^ ^]^ The dilution effect provided by carbohydrates is responsible for an observed reduction in the emission of formaldehyde during the binder cure process and within the final mineral wool product. This effect has been observed in PFU binders upon addition of sugar alcohols such as glycerol^[^ ^68^ ^]^ and upon the addition of starch or its degradation products.^[^ ^69^ ^]^ An alternative approach exploiting these reagents has been reported in which a secondary resin consisting of glycerol, dicyandiamide, and formaldehyde is generated separately and then added to a phenolic resole prior to spraying and curing; this secondary resin can also act as a fire‐retardant, in addition to scavenging residual formaldehyde.^[^ ^70^ ^]^

The addition of reducing sugars such as glucose to PFU binders for mineral wool has also been documented.^[^ ^47^, ^61^, ^71^ ^]^ Interestingly, the reduction in VOCs observed for reducing sugars cannot be fully explained by a dilution effect alone. Jobber et al.^[^ ^47^ ^]^ speculate that the reduction in VOCs is a result of a reaction between the carbonyl group of the reducing sugar with ammonia to form an imide. A related example describes a binder based on a combination of a PFU resin, a reducing sugar and a partially base neutralized styrene‐maleic anhydride (SMA) copolymer.^[^ ^43^ ^]^ This supposedly enables the final mineral wool product to achieve acceptable tensile properties with a lower mass of binder than the comparable control.^[^ ^72^, ^73^ ^]^

### 4.7 Other Scavenging Agents and Additives

Castro‐Cabado et al.^[^ ^53^ ^]^ recently disclosed that a wide range of peroxide‐containing oxidizing agents (e.g., hydrogen peroxide, ammonium persulfate, sodium percarbonate, etc.) can be added to PFU binders for MWI without deteriorating their cure time or mechanical properties (Figure 6, red circle). These molecules can undergo oxidation reactions with formaldehyde to form formate and formic acid at alkaline pH values, and reportedly reduced the free formaldehyde content of these resins by tenfold, to values <0.5 wt% as determined using photometry.

Although not acting in a capacity to reduce residual free formaldehyde, Didier^[^ ^74^ ^]^ has reported that the addition of carboxylic acid metal salts, especially magnesium phthalate and magnesium citrate, to phenolic binders for mineral wool improves their heat and punking resistance. The binders in this case had already achieved lower free formaldehyde values through the addition of urea and carbohydrates as described above.

### 4.8 Alternative Approaches

Foury and Noirbousson^[^ ^75^ ^]^ have outlined a method whereby formaldehyde emissions were reduced through the application of hydrazine carboximidamide sulfate onto the mineral wool once the binder has already been cured (Figure 6, yellow and red circles). It is thought that the hydrazine carboximidamide sulfate acts as a coating layer on the mineral wool, scavenging formaldehyde before it can escape to the surrounding environment. The use of hydrazine is challenging however, due to its own hazardousness, difficulty in handling, and cost.

Boyer et al.^[^ ^76^ ^]^ have also described an alternative scavenging process in which a separate backing sheet that carries a composition of formaldehyde scavengers can be placed adjacent to the fibrous mineral wool mat. This backing sheet is stated as consisting of paper, foil, a glass mat, or fabric that has been impregnated with aqueous solutions of sodium bisulfite, sodium metabisulfite, etc., and then dried. While shown to be highly effective as a scavenging technique, the preparation and assembly of the two separate components for this insulation product is expected to considerably increase the complexity of the manufacturing process.

Overall, through a careful combination of urea, various other formaldehyde scavengers, and/or changed manufacturing practices, it is possible to dramatically reduce formaldehyde emissions of PF resole‐based binders to levels considered acceptable by current indoor air quality regulations. In many cases these additives can also endow the PF binders with other beneficial properties, such as increased tensile strength and heat resistance. However, these binder compositions cannot be considered to be truly formaldehyde free, because it is impossible to economically reduce the total free formaldehyde within a binder to zero within the framework of the mineral wool manufacturing process. This issue is further compounded by the labile nature of urea‐containing binders and their tendency to release low levels of formaldehyde over time. PF resole binders also perform quite poorly in terms of sustainability because their feedstocks are heavily derived from petrochemicals.

## 5 Formaldehyde Free Binders for Mineral Wool Insulation

Trends in the regulation of free formaldehyde in the indoor environment, and marketability factors, have resulted in a steady shift in binder technology for MWI away from PFU resoles over the last few decades. Manufacturers are now investing heavily into the development of alternative binder chemistries that omit the use of formaldehyde altogether—so called “non‐added formaldehyde” or “substantially formaldehyde free” binders. These will be referred to here simply as “formaldehyde free” binders. For cost reasons, these novel binders are dominated by resins predominantly consisting of polyester bonds, but presently there are no thermosetting binder resins that can entirely outperform PFU resins on a cost‐for‐performance basis.^[^ ^55^ ^]^ Any novel binder technology would also ideally be a drop in replacement system, allowing manufacturers to use existing infrastructure and equipment, but in many examples this is not the case.^[^ ^77^ ^]^

Formaldehyde free binder alternatives to PFU resoles for MWI can generally be separated into four main categories based on the composition of their chemical feedstocks:polycarboxylates, polyvinyl alcohol (PVA), bio‐based, and epoxy resins. There are also numerous examples of binders for mineral wool where two or more of these categories have been combined in a single system. Of these categories, polycarboxylate binders were some of the earliest examples of formaldehyde free binders for non‐woven materials capable of competing with established PFU systems, and were first proposed as early as the 1950s.^[^ ^78^ ^]^ This was gradually followed by the disclosure of PVA binders for use in various systems, including insulation products. Both of these systems are still widely used today, but in recent years have begun to face increasingly stiff competition from bio‐based alternatives. Bio‐based binders for MWI were first used in the mid to late 2000s, and have since become one of the most heavily patented formaldehyde free binder technologies in the industry.

## 6 Polycarboxylate Binders

**Scheme 6:**
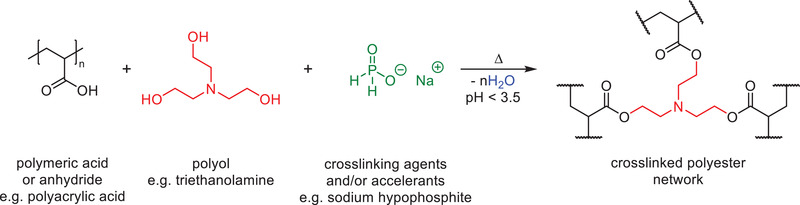
Typical poly(carboxylate) binder formulation.

The most common examples of this class of thermosetting binders are based on a macromolecular carboxylate and a low molecular weight polyol crosslinking agent. Due to the somewhat ambiguous nature of patents, the polymeric component will be referred to using the general term “poly(carboxylate)” herein, unless the precursor components are clearly stated. These polymer resins typically cure at high temperatures (>150 °C) under acidic conditions via the formation of ester bonds. The poly(carboxylate) component may be a homopolymer or copolymer prepared from unsaturated carboxylic acids, unsaturated anhydrides, or mixtures thereof. Common carboxylic acid monomers include acrylic acid, methacrylic acid, maleic acid, and itaconic acid, while common anhydrides include maleic anhydride and methacrylic anhydride. The polyol is typically a low molecular weight compound containing at least two hydroxyl groups, such as ethylene glycol, glycerol, glucose, and triethanolamine (**Scheme** **6**).^[^ ^79^ ^]^ There are also examples where commercially available macromolecular polyols, such as PVA, partially hydrolyzed polyvinyl acetate (PVAc) (e.g., ELVANOL), or mixtures thereof, have been used.^[^ ^80^ ^]^ These systems are described in the following section on PVA binders. β‐hydroxyalkyl amides (e.g., bis[N,N‐di(β‐hydroxyethyl)] adipamide) have also shown to be effective crosslinking agents^[^ ^81^, ^82^ ^]^ because they can undergo esterification reactions much faster than simple polyols in the absence of a catalyst.^[^ ^83^ ^]^

Poly(carboxylate) binders possess sufficient mechanical properties for applications in MWI, but generally suffer from high viscosities, among other issues. A lower viscosity, in addition to facilitating handling of the resin, e.g., by pumping, mixing, etc., also has an often beneficial effect on the properties of the mineral wool products obtained after curing. For instance, a low viscosity enables better penetration of the sprayed binder into the mineral fibers, improving homogeneity of the binder distribution. However, if the relative content of poly(carboxylate) is reduced the mechanical bonding properties of the cured binder begin to decrease considerably and the cure time increases.

There are two main approaches aimed at reducing viscosity: incorporation of low molecular weight species and the use of additional cross‐linkers or accelerants to allow for further dilution. Taylor et al.^[^ ^84^ ^]^ describe the use of a low molecular weight poly(carboxylate) while Strauss^[^ ^85^ ^]^ used a monomeric trihydric alcohol such as glycerol as the crosslinking agent to lower the viscosity. Care must be taken however because it has more recently been discovered that binder components comprising only glycerol as the alcohol reactant have an undesirably low smoke point (i.e., are prone to punking).^[^ ^86^ ^]^ Alternative polyols such as pentaerythritol can negate this issue but cannot be used on their own because they are solids at room temperature and increase binder viscosity. The solution is usually to employ a mixture of alcohols to balance these competing effects. The addition of urea has also been reported to greatly decrease the smoking of polyester binders during curing.^[^ ^87^ ^]^ Strauss and Williams^[^ ^81^ ^]^ report that the viscosity can also be reduced through the addition of a trifunctional monomeric carboxylic acid such as citric acid. Another approach is to take the pre‐reacted product of a poly(carboxylate) and a polyol and add it to the final binder composition.^[^ ^88^, ^89^ ^]^

Alternatively, Zhang^[^ ^90^ ^]^ used polyvalent metal compounds, for example calcium or zinc(II) oxide, to act as crosslinking agents. The metal compound is used in conjunction with a polyol to achieve a higher crosslink density post cure. The improved mechanical properties resulting from the higher crosslink density allows for a more diluted binder composition to be used whilst maintaining the mechanical properties, thus lowering the viscosity. In a similar manner, accelerants such as phosphorous containing salts and fluoroborates have been observed to increase the crosslink density of the cured binder, thereby enabling a lower initial viscosity.^[^ ^84^, ^91^, ^92^ ^]^ Lai et al.^[^ ^93^ ^]^ and later Anderson et al.^[^ ^86^ ^]^ reported that binders made using itaconic acid offer a similar improvement.

In most cases, these binders require longer cure times or higher temperatures in order to achieve a complete cure when compared to conventional PF/PFU binders, so manufacturing costs are higher. A method to alleviate this is to use a curing catalyst such as cyanamide, dicyanamide, or cyanoguanidine.^[^ ^94^ ^]^ Generally, binders formulations from poly(carboxylate)s and polyols are more hydrophilic than conventional PF/PFU resins, possibly compromising final product integrity. This risk can be minimized by incorporating a hydrophobic vinyl compound, e.g., styrene.^[^ ^84^, ^95^, ^96^, ^97^, ^98^ ^]^ For instance, Lundquist^[^ ^95^ ^]^ and Srinivasan et al.^[^ ^96^, ^98^ ^]^ have both described a binder composition based on a modified SMA copolymer, which has the additional benefit of being processed under alkaline conditions. Typically, binder compositions comprised of crosslinkable poly(carboxylate)s are formulated at highly acidic pH values, which poses a heightened risk of corrosion during manufacture and requires the use of more expensive equipment build from stainless steel.

In summary, poly(carboxylate) thermoset resins are effective binders for MWI products, but suffer from drawbacks arising from their reduced curing efficiencies and much higher viscosities in comparison to traditional PFU systems. The raw materials used to produce the poly(carboxylate)s are also relatively expensive and ultimately derived from fossil fuels. Their use is therefore a consideration of how much more the customer is willing to pay for a truly formaldehyde free product. Nevertheless, progress continues to be made to overcome these hurdles using the methods described above, as well as through the possible addition of various water soluble and cheap bio‐based extenders to these binder compositions, such as lignin, low molecular weight starch and soybean protein,^[^ ^99^ ^]^ examples of which will be discussed in further detail below.

## 7 Polyvinyl Alcohol Binders

**Scheme 7:**
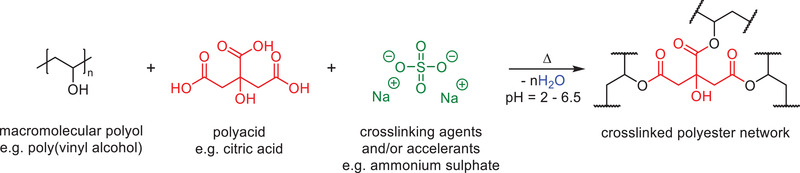
Example PVA binder formulation.

PVA is a water‐soluble polymer possessing desirable physical properties such as high tensile strength, excellent dimensional stability, non‐toxicity, and outstanding binding capacity. It is typically prepared via the hydrolysis of PVAc and its physical properties thus depend on the degree of esterification. PVA can be cured at high temperatures under acidic conditions in the presence of a crosslinking agent. The most frequently used crosslinking agents are multifunctional carboxylic acids, e.g., citric acid, which can react with alcohol moieties on PVA to create crosslinks in the form of ester bonds (**Scheme** **7**). In this way, the chemical functionality underpinning PVA binders is largely identical to that employed in poly(carboxylate) binders, but their roles are reversed. PVA has been used extensively within the paper and textile industries, but has traditionally been considered too viscous for use within MWI products. In cases where mineral wool PVA binders have been disclosed they are typically restricted to very dilute PVA concentrations between 1 and 5 wt%. PVA based binder compositions also suffer from similar flaws to poly(carboxylate) binders. These include longer cure times or higher cure temperatures, acidic conditions, and expensive petrochemical‐derived raw materials. Nevertheless, a handful of binder compositions have been developed to overcome these complications.

In an early example, Kajander and Bennett^[^ ^100^ ^]^ described the commercially available resin AIRVOL 205 as an effective MWI binder when diluted to a concentration of 4 wt% in water. AIRVOL 205 is comprised of partially hydrolyzed PVA polymers and contains low levels of VOC components. Pisanova et al.^[^ ^101^ ^]^ described an aqueous binder composition comprising a hydroxy‐containing polymer and a crosslinking agent in the form of a multifunctional carboxylic acid or anhydride. The hydroxy‐containing polymer is a combination of PVA and a starch, modified starch or sugar compound, which increases binder renewable content. The inventors claim that certain carboxylic acids, their salts or anhydrides, and even glucose can be dissolved in PVA solutions to reduce viscosity via hydrogen bond disruption and plasticization. An analogous approach instead describes binders in which PVA is combined with large amounts of an organooxysilane compound such as tetraethoxysilane.^[^ ^102^ ^]^ The authors report that such binders are stable for at least two weeks at room temperature, and at least two months when refrigerated (at ≈4 °C), and can be cured in 10 min at 180 °C. A likely drawback is the much higher relative cost of organooxysilanes.

**Figure 7:**
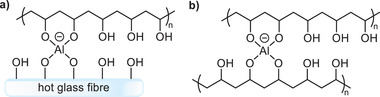
Coordination complex formation between metal salts and PVA, facilitating a) highly covalent bonding with glass fibers, and b) crosslinking between PVA chains.

Zhang et al.^[^ ^103^ ^]^ and later Smith et al.^[^ ^104^ ^]^ have both proposed aqueous binder compositions comprising a hydroxy‐containing polymer consisting of PVA and PVAc, a multifunctional carboxylic acid, in particular PAA, and low molecular weight polyols such as glycerol or sorbitol. It is thought that this binder acts as a hybrid system, with the low molecular weight polyol having an additional function as a secondary crosslinking agent. Metal salts, preferably either aluminum chloride, nitrate, or sulfate can also act as PVA cross‐linkers by forming coordination complexes between the polymer's hydroxyl functionalities.^[^ ^104^, ^105^ ^]^ This may be between adjacent PVA molecules, or between the PVA and the glass fibers themselves, with both cases providing additional strength to the resulting mineral wool (**Figure** **7**).

## 8 Bio‐Based Binder Compositions

**Scheme 8:**
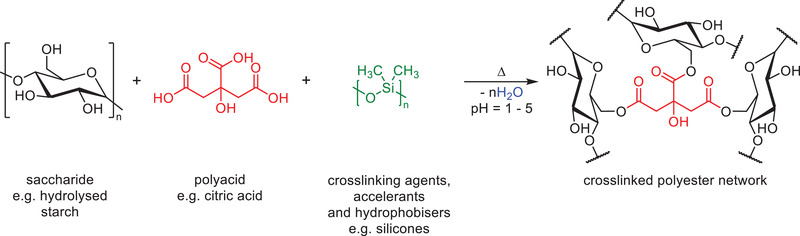
Example carbohydrate binder formulation.

Formaldehyde free binder materials based on renewable materials have been a recent focus within the mineral wool industry. Bio‐based binder compositions are generally cheaper than poly(carboxylate) and PVA compositions because of their reduced raw material costs. One of the most common binders is based on carbohydrates coupled with low molecular weight polycarboxylic acid or anhydride crosslinking agents (**Scheme** **8**). The carbohydrates used are mainly starch, modified starch, or sugars. These are commonly referred to in the literature as saccharides, meaning an organic compound containing sugar(s). Inorganic metal salts, for example, aluminum or copper sulfate, have also been disclosed as alternative crosslinking agents.^[^ ^90^, ^106^ ^]^ These carbohydrate binders cure at high temperatures under acidic conditions. The chemistry is similar to that of a PVA binder, whereby a polycarboxylic acid reacts with the hydroxyl groups of carbohydrates to form crosslinks in the form of ester bonds. The cure reaction is slower than conventional PF/PFU binder compositions, but can be accelerated in the presence of polyvalent metal oxides and various other additives.^[^ ^107^, ^108^ ^]^

### 8.1 Carbohydrate‐Based Binders

Water‐soluble carbohydrates are preferred in order to facilitate processing and curing reactions. Native starch is insoluble, does not impart adequate water resistance, and is generally considered too viscous for use within a binder composition. Starch can be modified (e.g., via hydrolysis) through chemical or enzymatic processes to improve its properties.^[^ ^109^, ^110^ ^]^ Such binders are also frequently extended with low molecular weight polyol additives such as glycerol, which also act as a means of controlling binder viscosity. Small amounts of esterification catalyst(s) are also typically added to the binder composition, and can include Lewis acids (e.g., silicates) and phosphorus‐containing compounds (e.g., sodium hypophosphite), among others.^[^ ^111^ ^]^

Hawkins et al.^[^ ^112^ ^]^ report a binder comprised of a modified starch and a citric acid or PAA cross‐linker. The starch is claimed to have a reduced viscosity and excellent resistance to water. Several others describe the use of reactive silicones and various other additives within carbohydrate and polycarboxylic acid binder compositions. The silicone acts as a hydrophobic barrier as in traditional PFU systems.^[^ ^113^, ^114^, ^115^, ^116^, ^117^ ^]^ Quinn^[^ ^118^ ^]^ has also disclosed a binder based on modified starch that instead makes use of various phosphorus based cross‐linkers such as phosphoric acid and phosphonic acid, etc. An alternative approach involves taking hydrolyzed starch and exposing it to a strong oxidizing agent such as hydrogen peroxide, to convert a portion of the secondary alcohol moieties into aldehydes.^[^ ^77^ ^]^ The resulting polyaldehydes were cured by reactions with diamines (e.g., l‐lysine) and showed improved mechanical properties.

Perhaps counterintuitively, Castro‐Cabado et al.^[^ ^119^ ^]^ have proposed a binder composition consisting mainly of insoluble starch (unmodified) and a poly(carboxylate) component such as acrylic acid. The inventors claim that the binder has an adequate viscosity despite the insoluble starch due to a dispersing and stabilizing effect exerted by the poly(carboxylate). Jaffrennou et al.^[^ ^120^, ^121^ ^]^ have described binder compositions based on modified or hydrogenated saccharides (e.g., corn starch) and polycarboxylic acid cross‐linkers that also contain at least one reducing monosaccharide, such as glucose. Two later patents also specifically mention that non‐reducing monosaccharides like fructose can function effectively in this role, possibly to avoid infringing on earlier work.^[^ ^122^, ^123^ ^]^ Another patent reports that the use of higher polyglycerols in such binders (e.g., diglycerol, triglycerol, tetraglycerol, etc.) provides improved mechanical properties by increasing binder crosslink density.^[^ ^124^ ^]^

Varagnat and co‐workers^[^ ^125^ ^]^ have developed a binder composition possessing enhanced tensile strength comprising of a saccharide, polycarboxylic acid and at least one polymer or copolymer of vinyl acetate, but requires highly acidic conditions (pH ≈ 1.5). Although not always so severe, acidic conditions are again a general requirement for carbohydrate binders, enabling crosslink formation and improving binder stability. In certain instances where there is a risk of metal surface corrosion during the service life of an insulation product, for example, in applications involving metal pipes or air ducts, there are reports of corrosion being counteracted by spraying these surfaces with aqueous solutions containing mild alkali salts (e.g., sodium carbonate) after the binder has been cured.^[^ ^126^ ^]^

### 8.2 Maillard Binders

**Figure 8:**
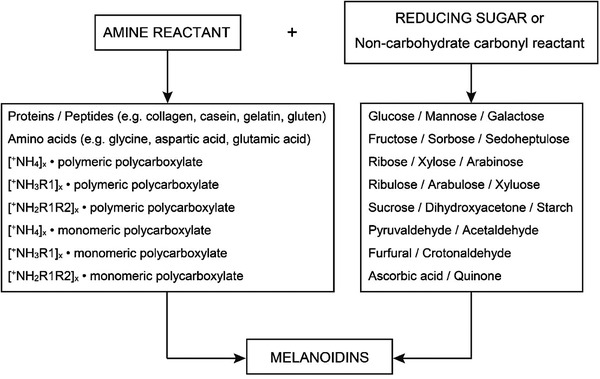
Common examples of amines and reducing sugar reactants used in the production of Maillard binders. Redrawn from ref. ^[^ ^130^ ^]^.

Another variant of bio‐based formaldehyde free MWI binders combines a carbohydrate, a source of nitrogen, and a carboxylic acid or poly(carboxylic acid) (or similar). Among other mechanisms, such binders are capable of curing via Maillard reactions that are traditionally of relevance in the food industry.^[^ ^127^ ^]^ The generalized Maillard process begins with a reaction between a reducing sugar, such as glucose, and a compound possessing a free amino group, to give a condensation product (**Figure** **8**).^[^ ^128^, ^129^ ^]^ This is proceeded by a range of reactions including cyclizations, rearrangements, isomerizations, and further condensations that ultimately lead to the formation of brown nitrogenous polymers and copolymers, referred to as melanoidins. Melanoidins are complex, high molecular weight species that vary in structure depending on the specific reactants and preparation conditions; for example, melanoidins display a degree of chemical aromaticity, unsaturation, and a C:N ratio that increases with heating time and temperature.^[^ ^129^ ^]^ MWI binders based on this broad class of chemical reactions have been patented extensively since the mid‐to‐late 2000s.

In perhaps the earliest example of a binder for MWI likely to undergo Maillard reactions, Husemoen et al.^[^ ^131^ ^]^ described formulations containing acids (e.g., PAA) and anhydrides (e.g., tetrahydrophthalic anhydride used in combination with amines (e.g., diethanolamine). Swift and co‐workers^[^ ^130^, ^132^ ^]^ later disclosed binders containing a reducing sugar, an ammonium salt of a polycarboxylic acid, and an optional source of nitrogen such as an amino acid or protein, making specific reference to the possibility of Maillard reactions. The ammonium salt triammonium citrate was particularly effective, providing both a carboxylate crosslinking agent and source of nitrogen. The binder can be formulated in alkaline conditions and the use of an ammonium acid salt is thought to reduce the risk of punking. In the majority of examples the curing oven temperature was set to ≈300 °C. Several other binder systems involving these or very similar reactants have been since been published by Swift,^[^ ^133^, ^134^ ^]^ Hampson et al.,^[^ ^135^, ^136^ ^]^ Jaffrennou and Roncuzzi,^[^ ^113^ ^]^ Shoemake and Breyer,^[^ ^137^ ^]^ and Zwaag.^[^ ^27^ ^]^ In many of these cases the amine component is a simple diamine or triamine, such as hexamethylenediamine or ammonia. For example, Shooshtari et al.^[^ ^138^ ^]^ and Eckert et al.^[^ ^139^ ^]^ describe binders comprised of glucose, hexamethylenediamine or monoethanol amine, and maleic anhydride, with hydroxyl cellulose acting as a viscosity modifier. Hjelmgaard and coworkers^[^ ^140^, ^141^ ^]^ have also demonstrated that the carboxylic acid component can be replaced with sulfur‐based acids (e.g., sulfamic acid), in particular ammonium sulfamate, or ascorbic acid (and its derivatives), which has a reasonably acidic 3‐hydroxy group instead of a carboxylic acid functionality.

Lee et al.^[^ ^142^ ^]^ have subsequently reported similar binders in which the ammonium salts instead contained phosphorus‐based anions, e.g. ammonium phosphate, also noting an improvement in punking and flame retardation. Others later described the use of water‐soluble formulations containing the reaction products of a carbohydrate paired with a polyamine, a polymeric poly(amine),^[^ ^143^ ^]^ or a poly(amino acid),^[^ ^144^ ^]^ with the authors noting an increased rate of cure in the poly(amino acid) case. A related disclosure reveals that binders of this nature can also be formulated to be substantially free of strong polycarboxylic acids that pose a corrosion risk, if an amino acid is combined with another source of nitrogen (e.g., ammonia in combination with dextrose and l‐lysine).^[^ ^145^ ^]^

Manville and Shooshtari^[^ ^146^ ^]^ describe a binder comprised of an amino‐amide intermediate and a reducing sugar containing an aldose or ketose group. The amino‐amide compound is formed through the reaction of an amine, preferably 1,4‐butadiamine or 1,6‐hexadiamine, and a carboxylic acid or anhydride.^[^ ^147^ ^]^ The amine can also be the salt of an organic acid, which simultaneously reduces the risk of punking in the final MWI product.^[^ ^148^ ^]^ Analogous binders based on non‐reducing sugars have also been proposed.^[^ ^149^ ^]^ Savonnet et al.^[^ ^150^ ^]^ have also shown that commonly used diamines (e.g., 1,6‐hexadiamine) can be replaced with water soluble polyetheramine oligomers, generally referred to as Jeffamines. Very recently, Hampson and Callaghan^[^ ^151^ ^]^ unveiled Maillard binders based on cellulose hydrolysate sugars that are comprised of complex and variable mixtures of saccharides, including glucose, fructose, sucrose, and many others. These hydrolysate sugars were cured in the presence of inorganic salt cross‐linkers that can also act as a source of nitrogen (e.g., diammonium phosphate), and benzoic sulfimide can be added to further enhance the curing rate.^[^ ^152^ ^]^

Another series of Maillard binder systems for MWI products are based on the use of sugar syrups (e.g., molasses). The use of molasses can theoretically reduce the number of separate ingredients required by acting as a source of reducing sugars and functional amine compounds required for the Maillard reaction. In one example by Pacorel and Hampson,^[^ ^153^ ^]^ the binder also incorporates both polymeric and monomeric polycarboxylic acid components to form a composite that includes both melanoidin and polyester structures. The curing rate is controlled via a sodium hypophosphite catalyst and the binders exhibit excellent mechanical properties and weather resistance. Another example is based on corn syrup comprising at least 30% dextrose, an amino sugar such as glucosamine, and ammonium salts of inorganic acids (e.g., ammonium phosphate monobasic, etc.).^[^ ^154^ ^]^ These properties could be enhanced further still via the addition of a Lewis acid such as zinc sulfate. Hansen^[^ ^155^ ^]^ has also reported mineral wool binders from various sugar syrups including molasses crosslinked with citric acid that contain an additional nitrogen source (e.g., aqueous ammonia).

**Figure 9:**
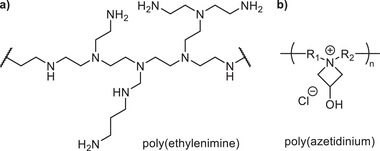
a) Example fragment of branched poly(ethylenimine). b) Example structure of the poly(azetidinium) polymers described by Hampson and Khan.^[^ ^157^, ^158^ ^]^.

Several recent examples of Maillard binders disclose the partial or complete substitution of simple difunctional amines and amino acids with synthetic polyamine components. For instance, Pacorel^[^ ^156^ ^]^ published a binder comprised of a polymeric product of at least one carbohydrate component, such as a reducing sugar (e.g., glucose), a polycarboxylic acid, and a branched poly(ethylenimine). Binders containing poly(ethylenimine) were shown to exhibit improved properties such as excellent curing rates, longer shelf lives, and high internal bond strengths of the final insulation products, likely due to the large number of interconnected amine sites available for further crosslinking (**Figure** **9**a). Hampson and Khan^[^ ^157^, ^158^ ^]^ have developed binders from mixtures of mono‐ and/or polysaccharides with polycarboxylic acids, alongside a class of ionic polyamine cross‐linkers known as poly(azetidiniums) (Figure 9b). Poly(azetidinium)s are highly reactive species on account of their ionic nature and four membered strained rings that can react with various functional groups through ring opening reactions, including those of saccharides.^[^ ^159^ ^]^ In some cases the binders are reported to show improved properties, including less undesirable coloration and improved mechanical properties. Endres et al.^[^ ^160^ ^]^ have also developed Maillard MWI binders that incorporate between 1 and 5 wt% of a polyamidoamine resin containing azetidinium functionalities, thereby drastically improving their wet tensile strengths.

In summary, carbohydrate‐polyamine binder compositions have faster cure times than the likes of poly(carboxylate)s or simple carbohydrate binder compositions. They can be formulated under alkaline conditions and exhibit mechanical properties that can compete with conventional PF/PFU binders. On the downside, while formaldehyde emissions are negligible or non‐existent, emissions of ammonia can be quite high; as with many other formaldehyde free binders, the acidity of certain components and associated corrosion of machine parts, particularly during curing, can also be an inherent problem. It is now known that saccharide binders can also generate furfural and/or hydroxymethylfurfural in the course of the preparation and/or curing process, both of which are also subject to strict regulatory controls, but can be minimized through careful binder formulation.^[^ ^161^ ^]^ Insulation products created using Maillard reactions typically have an undesirable dark brown color after curing and the use of large quantities of amines (e.g., ammonia) in the formulation presents a safety risk and possible emission problems.^[^ ^86^ ^]^

Another challenge associated with almost all binders that are at least partially bio‐derived, especially those based on hydrogenated sugars, is that the curing process must be more precisely controlled than for traditional PFU binders.^[^ ^162^ ^]^ Finally, as with many other binder systems, a challenge associated with the use of certain binders based on Maillard reactions is that the components of the precursor solutions are prone to “aging” by undergoing deleterious polymerization and/or crosslinking reactions prior to use. Hampson et al.^[^ ^163^ ^]^ have recently reported that these reactions can be retarded by carbamate compounds that are formed when carbon dioxide is bubbled through the precursor solution after it is freshly prepared. Limited storage life is also a problem commonly encountered in the use of pre‐reacted PFU binders, meaning that bio‐based binders not requiring pre‐reactions can actually be easier to handle in some cases.

### 8.3 Non‐Carbohydrate Bio‐Based Binders

**Figure 10:**
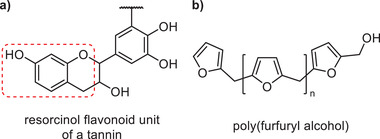
a) Flavonoid unit structure of tannin based on the resorcinol group (highlighted in red). b) Chemical structure of poly(furfuryl alcohol).

Several other notable bio‐based binder compositions that do not fit into the general cases outlined above have also been reported recently, suggesting that manufacturers are beginning to look to alternative cheap biomacromolecule feedstocks with thermosetting properties. Williamson and Jing^[^ ^164^ ^]^ have invented mineral wool binders that cure through reactions between (one or more) tannins (e.g., from black wattle) and multifunctional aldehydes (e.g., glyoxal) in the presence of Lewis acid catalysts (e.g., sodium silicate). Tannins are a group of water‐soluble phenolic compounds with a molecular mass between 0.5 and 15 kDa that have the ability to induce the precipitation of proteins and alkaloids (**Figure** **10**a).^[^ ^165^ ^]^

Salomon et al.^[^ ^166^ ^]^ described a binder composition based on water‐soluble poly(furfuryl alcohol) and an acid catalyst. Poly(furfuryl alcohol) is obtained by the treatment of biomass and is a well‐known resin precursor (Figure 10b).^[^ ^167^ ^]^ This follows similar chemistry to PVA binder compositions and can be thought of as a bio‐based alternative. Potential VOCs emanating from this binder composition may cause concern since poly(furfuryl alcohol) is designated harmful to humans if inhaled.^[^ ^168^ ^]^

In disclosures by Hjelmgaard^[^ ^169^ ^]^ and Hjelmgaard and Tielemann,^[^ ^170^ ^]^ binders comprising at least one phenol and/or quinone containing compound and at least one protein are proposed. The phenol/quinone compound is selected from a group of tannins obtained from natural sources such as oak, while the protein component is derived from animal (e.g., gelatine) or plant sources (e.g., soy protein). It is also possible to use enzymes (e.g., transglutaminase) to substitute the function of the phenol/quinone compound.^[^ ^171^ ^]^ Transglutaminase enzymes are known to catalyze transamidation reactions between the side chains of glutamine and lysine residues of gelatine to produce strong amide linkages.^[^ ^172^ ^]^ The claimed advantages are that these binders are non‐toxic, show improved aging resistance, and can be cured at significantly lower temperatures (i.e., ≈25 °C) than most other binder compositions.^[^ ^171^, ^173^ ^]^ In a related example, binders based on various whey protein feedstocks are reported.^[^ ^174^ ^]^ Whey is the liquid material created often as a by‐product of cheese production, and is comprised of proteins that typically have molecular weights >10 kDa and are able to polymerize through disulfide bond formation.^[^ ^175^ ^]^ In the most successful examples presented, the binders consist of an acid hydrolyzed whey protein source dispersed in 0.1 m NaCl at a concentration of 40 wt%, along with small quantities of curing accelerators, such as K_2_CO_3_ and CaCO_3_, and surfactants. These binders were found to gel in ≈2–3 min at 100 °C.

## 9 Epoxide Binders for Mineral Wool Insulation

**Scheme 9:**
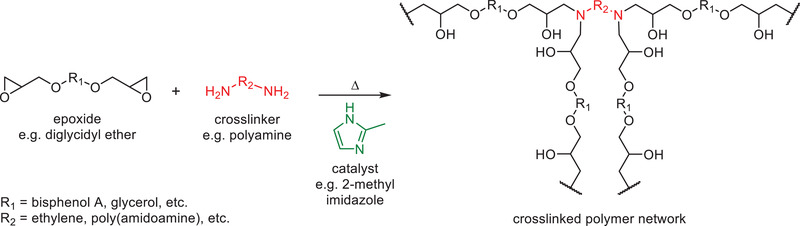
General curing reaction of an epoxide thermoset binder.

Epoxides represent one of the most prolific classes of thermosetting resins in the world, yet only a handful of patents describing their use as binders in MWI products have been published over the last few decades. The practical use of such binders has largely been limited by a combination of their higher costs, low heat resistance, low stability, and the limited water solubility of the most common precursors (e.g., bisphenol A diglycidyl ether). Nevertheless, the performance to cost ratio of epoxy resins is among the best of all known thermosetting materials, and their application potential continues to increase through the use of cheap fillers and novel chemistries.^[^ ^176^ ^]^ Epoxide binders typically exploit ring opening reactions between cheap diglycidyl ether molecules (e.g., glycerol diglycidyl ether) and polyamine cross‐linkers in the presence of imidazole‐based catalysts (**Scheme** **9**).

In an early example, Jackson et al.^[^ ^177^ ^]^ described formaldehyde free mineral wool coated with a sprayable epoxy resin binder containing at least one fire resistance additive (e.g., boric anhydride). The binder consisted of a mixture of a phenol component such as bisphenol A, an aliphatic epoxy resin such as polyoxyalkylene diglycidyl ether, and a trialkoxy boroxine curing agent. Another early example discloses water dilutable or dispersible mixtures of a diglycidyl ether of a polyol (e.g., bisphenol A diglycidyl ether), and a poly(amidoamine) crosslinking agent hardener formed from a reaction between diethylenetriamine and adipic acid.^[^ ^178^ ^]^ Espiard et al.^[^ ^179^ ^]^ developed similar binders that substitute the poly(amidoamine) with low molecular weight polyamines such as tetraethylenepentamine, in combination with the accelerator 2‐methylimidazole. It is also possible to obtain epoxide‐based binder precursors from natural sources through the epoxidation of various natural vegetable oils, preferably linseed oil and soybean oil.^[^ ^180^ ^]^ The highly acidic pH values (<3) required to achieve optimal binder strength is again a common drawback of these systems.

A more recent example makes use of mixtures of various high molecular weight (>50 kDa) water soluble polymers (e.g., PVA, polyvinylpyrrolidone), with lower molecular weight (4–12 kDa) water soluble polyethers (e.g., polyethylene glycol) possessing epoxy‐functionalized end groups.^[^ ^181^ ^]^ Alavi^[^ ^108^ ^]^ later described binders containing the epoxide molecules described above (e.g., bisphenol A diglycidyl ether and/or epoxidized plant oils) in combination with the components required for Maillard reactions, including carbohydrates such as modified starch, and a carboxylic acid ammonium salt such as triammonium citrate. A very recent patent makes use of the epoxides isosorbide diglycidyl ether and glycerol diglycidyl ether, in combination with cross‐linkers such as citric acid, sorbitol and maltitol, with the catalyst 2‐methylimidazole.^[^ ^182^ ^]^ The use of less reactive polyol and polyacid cross‐linkers greatly increased binder stability (i.e., gelation) at 25 °C, while still having controlled gel temperatures between 90 and 175 °C. Furthermore, the binders had a low viscosity regardless of the nature of the cross‐linker and in most cases could be formulated at a neutral to basic pH to minimize corrosion.

## 10 Conclusions

In conclusion, traditional PFU thermoset binders used in the manufacture of MWI products are rapidly becoming undesirable because free formaldehyde is released during their manufacture and service lifetime into atmospheres breathed directly by humans. Over the past two decades in particular, manufacturers have invested heavily in the development of novel binder systems for MWI products with very low formaldehyde emissions, or preferably, that omit the use of formaldehyde altogether. The commonly practiced strategy of modifying PFU binder formulations with so called “scavenging” molecules that ultimately reduce the amount of formaldehyde released during and/or after the thermal curing process has shown great success in this regard. In addition to carbamides (e.g., urea), common examples of such scavengers include ammonia, dihydroxybenzenes, reducing sugars, sodium bisulfite, alkanolamines, and others. In some cases these additives can also endow the PFU binders with other beneficial properties, such as increased tensile strength and heat resistance.

In switching to formaldehyde free thermosetting binder chemistries, manufacturers have largely opted to use thermosets formed through condensation reactions involving polyacids, polyols, and polyamines derived from various feedstocks with multifunctional cross‐linkers, on account of their relatively low costs. Alternatives based on higher performance yet high cost epoxide binders have also received some interest. Nevertheless, typical examples of these binders struggle to outperform PFU resins on cost per performance metrics due to the various additional challenges associated with their use, including insufficient mechanical properties, high viscosities, low stabilities, unsuitable curing properties, corrosion, and incompatibilities with existing manufacturing equipment. The underlying chemistry of PFU systems is also very well understood and the industry has largely been developed and optimized around them, creating additional perceptual barriers to new binder adoption.

The patents described herein reveal that manufacturers have sought to exploit various other organic, inorganic, and polymeric additives to overcome each of these issues, including cross‐linkers, catalysts, cheap bio‐based feedstocks and fillers, viscosity and pH modifiers, corrosion inhibitors, and many others. Ultimately, tremendous progress has now been made by MWI manufacturers to identify formaldehyde free binders that do not sacrifice function. Formaldehyde free binders are now being ever more widely adopted by the industry and it appears to only be a matter of time until PFU binders are rendered completely obsolete.

## Conflict of Interest

The authors declare no conflict of interest.
